# First Australian Case of Good Recovery of a COVID-19 Patient With Severe Neurological Symptoms Post Prolonged Hospitalization

**DOI:** 10.7759/cureus.10366

**Published:** 2020-09-10

**Authors:** Tissa Wijeratne, Carmela A Sales, Sheila G Crewther, Vinh Nguyen, Leila Karimi

**Affiliations:** 1 Department of Neurology, Australian Institute for Musculoskeletal Science, Western Centre for Health Research & Education, Melbourne Medical School, Sunshine Hospital Western Health, St Albans, AUS; 2 Department of Psychology and Public Health, La Trobe University, Bundoora, AUS; 3 Department of Medicine and Dean's Office, University of Rajarata, Saliyapura, LKA

**Keywords:** covid-19, acute ischemic stroke, microcirculaiton and inflammation, neutrophil to lymphocyte ratio (nlr), c-reactive protein, d-dimer, mrf-neural, slurp, neurorahabilitation, severe neurological manifestation

## Abstract

A case of a 75-year-old man with COVID-19, severe neurological symptoms (acute stroke-like symptoms and signs and full recovery after a prolonged hospital stay), and intracranial hypertension is discussed with an in-depth review of his clinical features, biochemistry, haematology, highlighting the relationship between changes in neutrophil-lymphocyte ratio, C-reactive protein level, D-dimer level, and the clinical onset of acute ischemic stroke-like symptoms in the setting of COVID-19 and major neurological manifestations. This is the first such case reported in Australia to date. This case also illustrates the recovery of a patient with COVID-19 complicated with severe neurological symptoms (acute ischemic stroke-like symptoms) during the prolonged intensive care unit stay (at day 26) followed by slow neurorehabilitation and normal recovery from both respiratory and neurological involvement. The onset of acute stroke-like symptoms appears to be closely associated with changes of neutrophil-lymphocyte ratio and in C-reactive protein, and D-dimer levels.

## Introduction

In December 2019, a novel coronavirus associated with a series of atypical pneumonia was first detected in Wuhan, China. Since then the virus, now known as severe acute respiratory syndrome coronavirus - 2 (SARS-CoV-2), has spread to over 216 countries and is now recognized as a significant global pandemic of COVID-19. As of 4th August 2020, the total number of COVID-19 cases recorded worldwide was 18,456,665 with the number of confirmed deaths at 697,435 [1].

Neurological manifestations of COVID-19 were first reported in Wuhan, China, where 5% of total reported cases also suffered an acute ischemic stroke (AIS) [2]. By comparison, the rate of AIS in association with COVID-19 in New York, USA, was only 0.9% [3]. Several theoretical models have been proposed to explain the occurrence of neurological events among patients with COVID-19 with most focusing on COVID-19-induced severe inflammation (and endothelial dysfunction) and activated prothrombotic pathways resulting in microthrombosis [4]. We describe the first case of COVID-19 with AIS-like severe neurological manifestation in Australia with unusual neutrophil-lymphocyte ratio (NLR) occurrence presumably indicative of the onset of severe neurological symptoms (acute ischemic stroke-like symptoms) during the day 21-26 of the illness. 

## Case presentation

**Figure 1:**
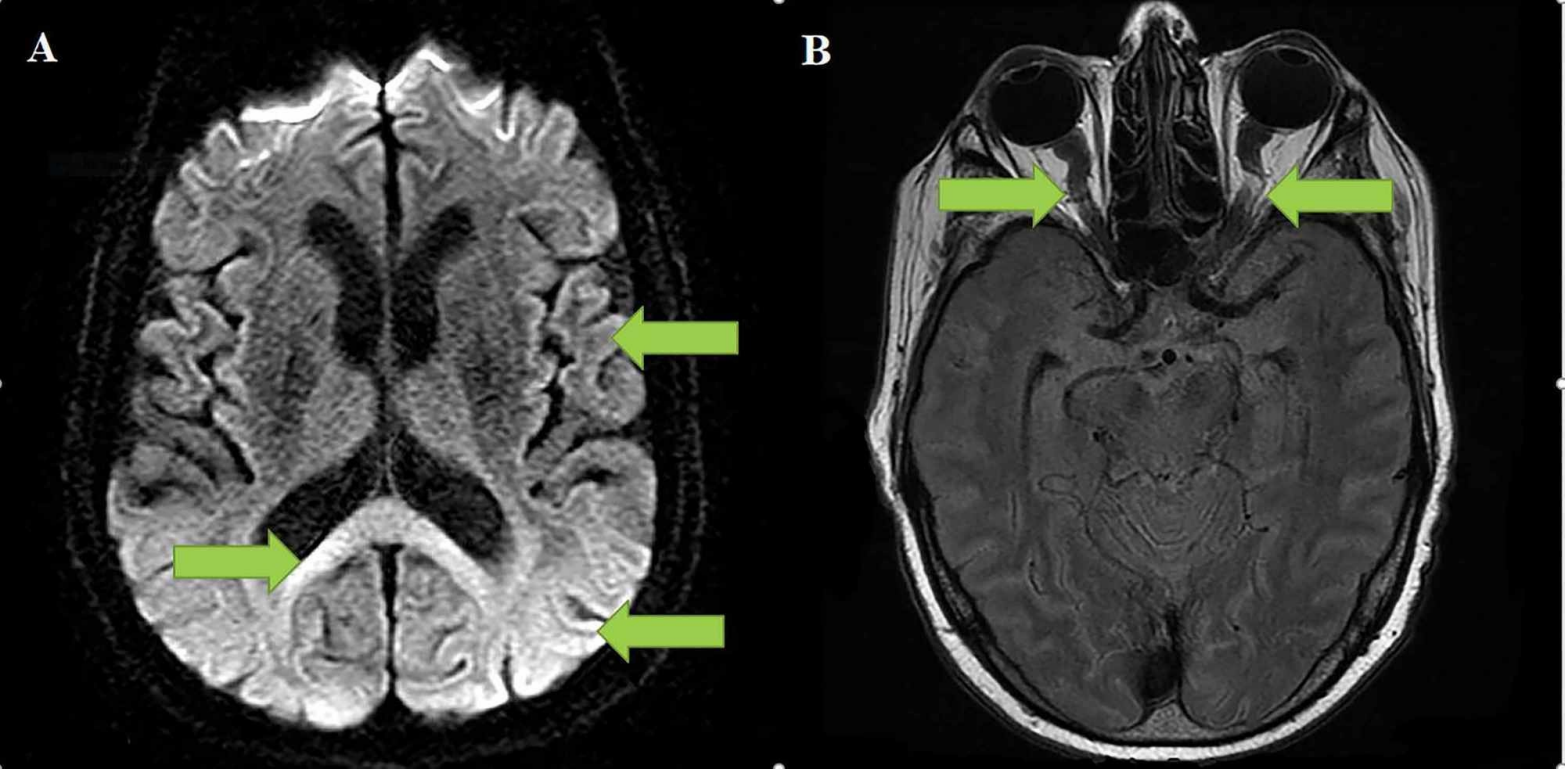
1A demonstrates severe diffusion restriction in the brain's subcortical regions and the splenium; 1B demonstrates the tortuous optic nerve sheath secondary to intracranial hypertension Arrow A demonstrates the severe diffusion restriction throughout the subcortical region and the splenium. Arrow B demonstrate the tortuous optic nerve sheath secondary to intracranial hypertension.

A man hospitalized with COVID-19 developed significant neurological manifestations (acute stroke-like symptoms) between 21-26 days post infection which were later confirmed with magnetic resonance imaging (MRI), showing changes typical of raised intracranial pressure and significant diffusion restriction suggestive of cytotoxic or excitotoxic, transient oedema secondary to inflammation and severe hypoperfusion state (Figures 1A and 1B).

The 75-year-old male patient was managed at an Australian metropolitan tertiary care hospital. SARS-CoV-2 infection was confirmed in this patient by reverse transcriptase polymerase-chain-reaction (RT-PCR) assay on admission to the ED. Currently, this appears to be the first COVID-19 case with severe neurological manifestations (acute ischemic stroke-like symptoms and raised intracranial pressure) in Australia to date.

**Figure 2:**
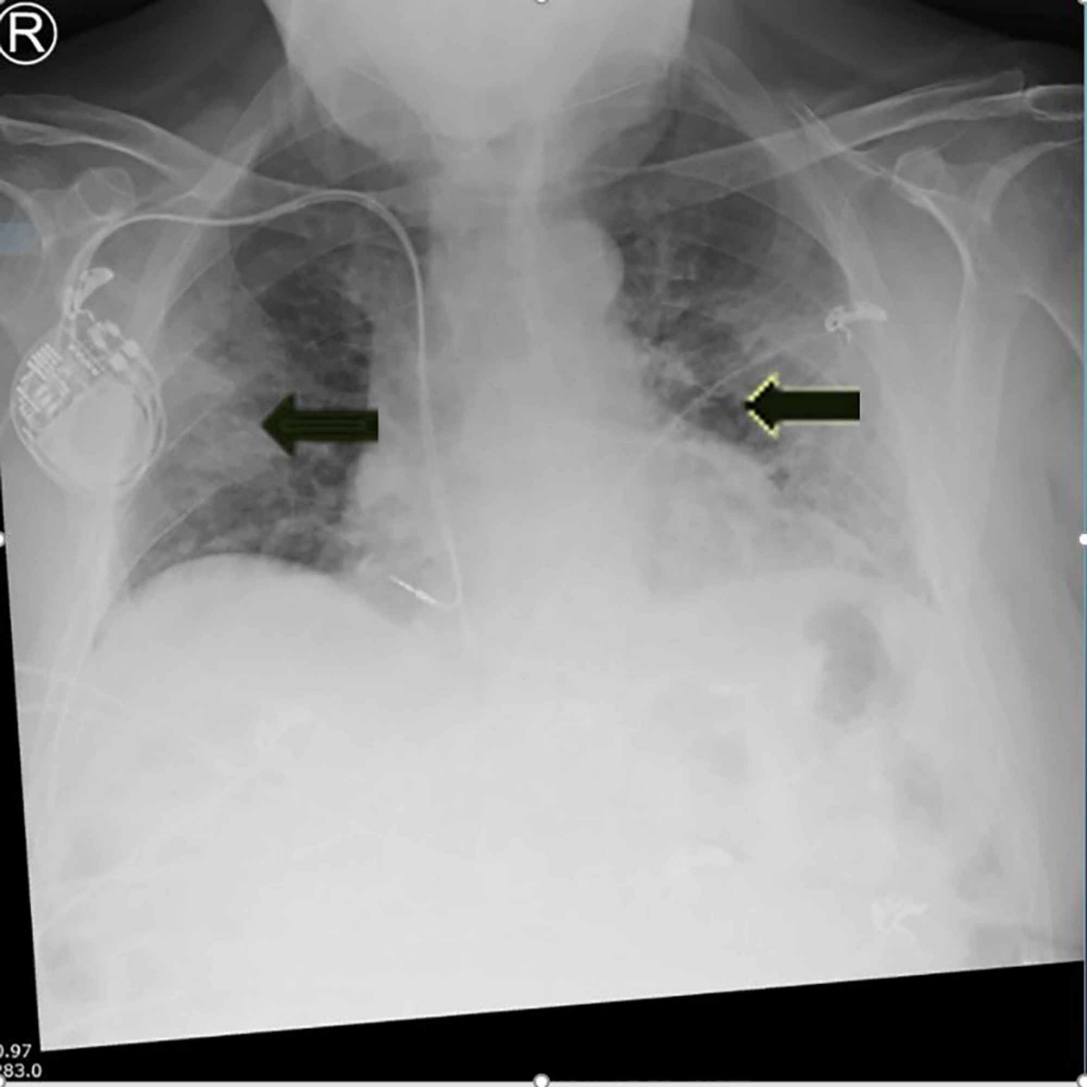
Bilateral chest X-ray on admission showing basal opacities secondary to COVID-19-associated pneumonia Arrowheads demonstrate the bilateral, patchy opacities in the lungs

The previously well man reported a past medical history of asymptomatic and stable prostate cancer, as well as treated melanoma five years ago. He presented with a five-day history of fever, cough, shortness of breath, anorexia and sore throat to the ED in late March 2020. The patient had not travelled overseas and had no history of contact with any known case of COVID-19. He was febrile on admission with a temperature at 38.2° C, displaying slight tachypnoea (respiratory rate of 22) and a blood oxygen saturation of 95%. His blood pressure and heart rate remained stable upon arrival at the ED. His initial chest X-ray revealed patchy opacities in the mid to lower lung fields (Figure 2), indicating a likely diagnosis of COVID-19.

He was isolated for suspected COVID-19 infection in a general medical ward. Over the next 24 hours, the patient’s blood pressure started to drop in association with increasing hypoxia and worsening respiratory condition. Despite high-flow oxygen support, the laboured breathing persisted, warranting the initiation of invasive mechanical ventilation and the patient’s subsequent transfer to the intensive care unit (ICU). His first 48 hours in the intensive care unit (ICU) were complicated with further blood pressure drop and poor respiratory function. He remained on prophylactic anticoagulation (enoxaparin 40 mg/SC) from the onset of admission to prevent deep vein thrombosis (DVT). He remained RT-PCR positive for SARS-CoV2 until at least the 14th hospital day. Extubation was unsuccessfully attempted on the 19th ICU day but failed, necessitating reintubation within 24 hours. During this time, on the patient’s 24th hospital day, progressive right upper limb swelling became apparent and a clinical diagnosis of DVT was confirmed with an ultrasound showing extensive, non-compressible thrombi involving the right cephalic, cubital, basilic, axillary, subclavian and the internal jugular veins. For treatment of DVT, he was started on therapeutic anticoagulation with low molecular weight heparin (a full therapeutic dose of enoxaparin). He was noted to be significantly drowsy and less responsive over the next 72 hours. A referral to the neurology department was made due to the non-improvement of his altered conscious state despite pausing IV sedation during the 26th day of his admission. Initially the patient was thought to be displaying hypoxic encephalopathy secondary to a blood pressure drop or an acute stroke secondary to hypoperfusion or large vessel disease related to COVID-19.

**Figure 3:**
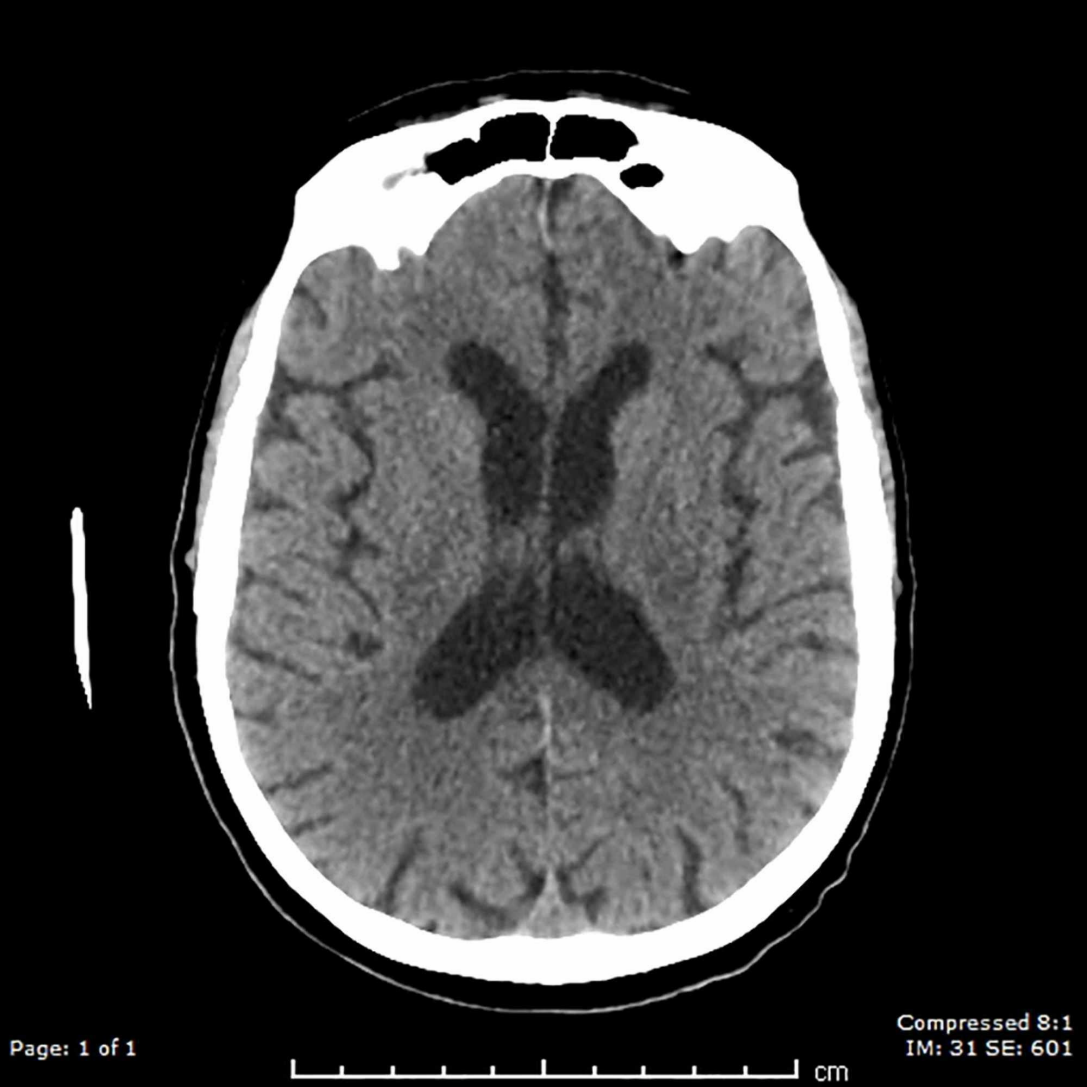
CT brain demonstrating normal findings Normal CT scan of the brain showing severe diffusion restriction of the MRI brain. The normal recovery after the prolonged hospital stay confirmed it was unlikely that the severe neurological and stroke-like symptoms were due to a stroke.

Neurological assessment on day 26 revealed a stuporous patient (Glasgow Coma Scale fluctuating between 3-6) with no physical signs of cranial nerve involvement. In particular, the patient showed no signs of gaze palsy or ptosis. Papilledema was excluded by fundoscopy while light reflex remained intact. There were no meningeal signs such as neck stiffness or a positive Kernig’s sign. He underwent tracheostomy on day 27 as he was not improving and pneumonia symptoms were worsening. He underwent a CT scan of the brain on day 28, showing no discernible structural abnormalities (Figure 3).

**Figure 4:**
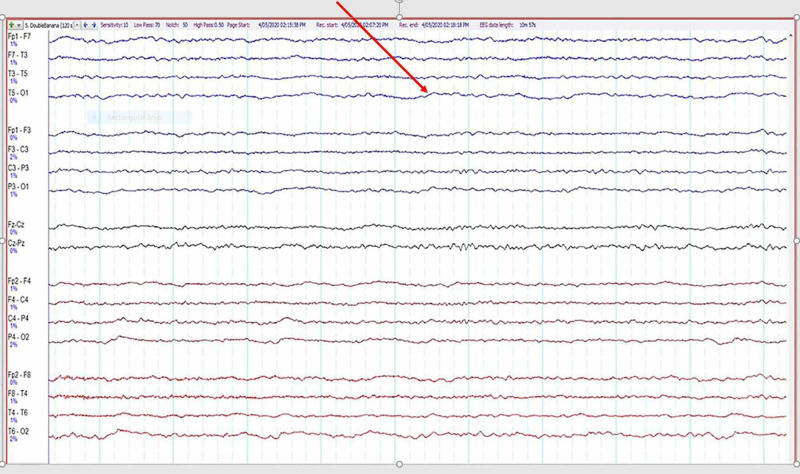
EEG showing symmetrical slow-wave activity secondary to cerebral involvement of COVID-19

 An EEG was performed on Day 30, demonstrating mild generalized slowing based on theta and intermittent delta wave activity (Figure 4).

Pertinent laboratory results on admission and during ICU stay are summarized in Table 1.

**Table 1:** Clinical parameters * denotes values that exceed normal reference ranges for a healthy male adult.

Parameter	Results (Range)	Normal Range in our Lab
Hemoglobin (g/L)	94.96 (67-137)*	130-180
White Cell Count (x10^9/L)	13.53 (7.2-23)*	4.0-11.0
Platelet count (x10^9/L)	341.09 (113-611)*	150-450
Neutrophil (x10^9/L)	2.08 (1.5-18.2)*	2.0-8.0
Lymphocyte (x10^9/L)	2.08 (1.5-3.2)	1.0-4.0
CRP (mg/L)	130.74 (19-361)*	<10
Na (mmol/L)	145.38 (134.57- 148.58)*	135-145
K (mml/L)	4.17 (3.8-4.7)	3.5-5.2
Urea (mmol/L)	14.63 (4.3-27)*	3.0-10.0
Creatinine (umol/L)	80.07 (45-172)*	60-110
Ionized Calcium (mmol/L)	1.29 (1.03-1.47)*	1.15-1.30
Magnesium (mmol/L)	0.99 (0.93-1.14)*	0.60-1.10
Phosphate (mmol/L)	1.05 (0.61-1.87)*	0.75-1.50
Alb (g/L)	22.83 (15-35)	34-47
Fibrinogen (g/L)	6.38 (4.2-8.2)*	1.5-4.0
Creatinine Kinase (U/L)	326.75 (32-747)*	0-240
D-dimer	1.11*	<0.50

The laboratory profile of this patient suggests that a generalized immune response to SARS-CoV-2 was apparent early, based on the neutrophil-lymphocyte ratio (NLR) and CRP, followed by persistent anemia, leukocytosis and hypoalbuminemia during their hospital stay. Urea, ionized calcium and CRP were also persistently elevated, while D-dimer, serum fibrinogen, and creatinine kinase peaked during admission but normalized subsequently.

An MRI of the brain was performed on day 32 that showed generalized cortical diffusion restriction, which was also present in the supra- and infratentorial white matter (Figure 1A).

A few scattered microbleeds in the frontal, parietal and temporal lobe were described along with a small volume subarachnoid haemorrhage in the right frontal lobe (not shown). There was no pachymeningitis noted, and neither were there any signs of venous nor arterial occlusion. The same MRI also revealed the typical features of intracranial hypertension in the appearance of optic nerve sheath distension and tortuosity (Figures 1A-1B) and transverse sinus narrowing and empty sella turcica (not shown in image). No intracranial masses were noted.

**Figure 5:**
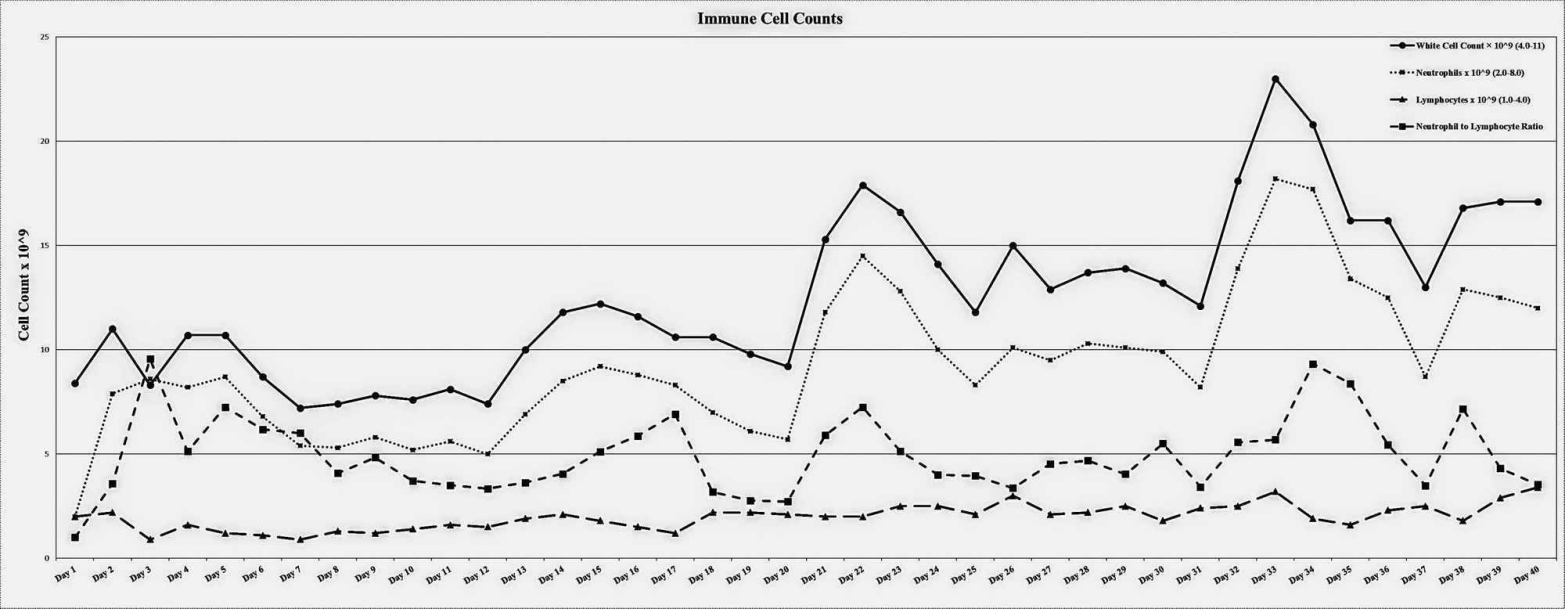
Immune cell graph Note the rapid rise in the neutrophil-lymphocyte ratio preceding the onset of stroke-like symptoms.

He was suspected as having an acute ischemic stroke secondary to severe hypoperfusion at this point of time. In terms of useful information, the patient’s immune profile included white cell counts, neutrophil counts and the neutrophil-lymphocyte ratio (NLR) peaked around this time. It is unlikely he had a stroke given the eventual complete recovery despite the prolonged hospital stay. His stroke-like symptoms and radiological changes were likely related to the COVID-19-related severe inflammatory changes and cytotoxic oedema. Data are illustrated in Figure 5, which highlights the rapid increase in white blood cell types immediately preceding the diagnosis of major clinical events such the severe neurological involvement (stroke-like symptoms) noted on day 26 and the patient’s worsening respiratory function during day 32. The stroke-like symptoms most likely occurred on the 21st day of ICU hospitalization). Figure 5 shows a similar pattern of perturbation of all haematology tests including NLR and falls in lymphocyte to C-reactive protein (LCRPR) and changes in liver and renal function indicative of a severe systemic inflammatory response over the first three weeks before the onset of acute stroke-like symptoms. Levels of urea, ionized calcium and CRP were also persistently elevated, while the levels of D-dimer, serum fibrinogen and creatinine kinase rose during admission but normalized subsequently. The significant delay in the patient’s referral to the neurology unit/team and a similar delay in the neuroimaging of the brain both indicate the possibility for underdiagnosis of stroke and other neurological disorders during this pandemic and inadequate specialists being available.

**Figure 6:**
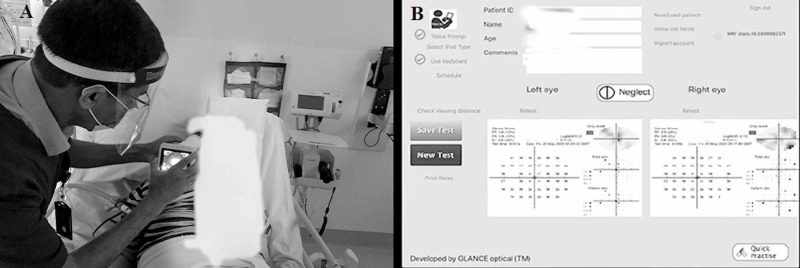
Functional vision tests at the bedside 6A: Retinal photography at the bedside using the RV100-B RetinaVue™ 100 Imager Non-Mydriatic Retinal Camera 6B: The Melbourne Rapid Fields-neural assessment, showing normal visual fields.

To assess the patient’s cognitive functions during rehabilitation and post neurological manifestations, retinal integrity and vision and visuomotor abilities were assessed at the bedside on the 58th-day post-hospital admission. Optic nerve head and retinal blood vessels integrity were found to be normal via fundus photography using an RV100-B RetinaVue™ 100 Imager Non-Mydriatic Retinal Camera (Welch Allyn, Skaneateles Falls, New York) (Figure 6A). Furthermore, the patient’s normal visual acuity and visual fields were tested with an iPad- based visual field perimetry application, Melbourne Rapid Fields - neural (MRF-n) [5-10] (Figure 6B).

**Figure 7:**
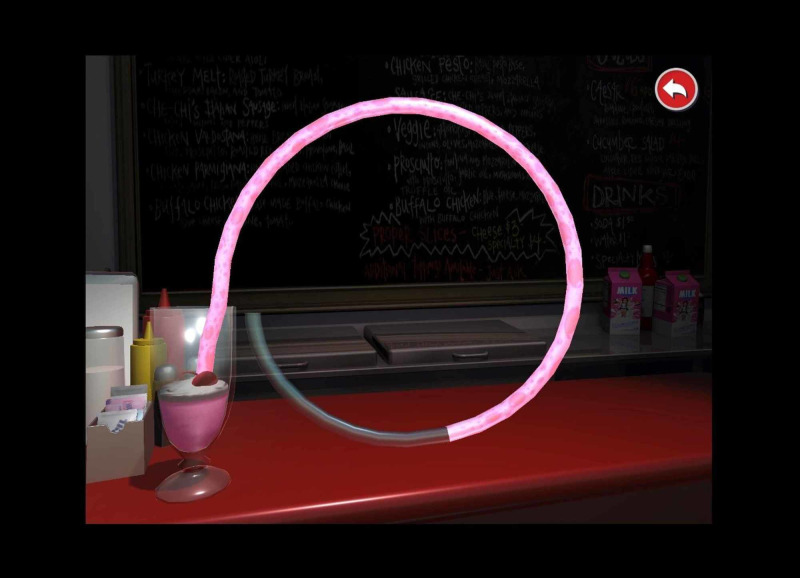
Lee Ryan Eye Hand Co-ordination Test (SLURP)

However, on visuomotor tasks, the patient took significantly more time than controls of similar age. His visual acuity and visual fields were tested in less than eight minutes in the ward with the help of the MRF-n, and was found to be normal [1-6] (Figure 2A-2B). The patient was also asked to perform three short visuomotor tasks borrowed from the Lee Ryan Eye Hand Co-ordination Test battery (SLURP)] as a rapid (less than five minutes) baseline measure of fine visually driven motor and executive function skills (Figure 7).

His immune profile including white cell counts, neutrophil counts and neutrophil lymphocyte ratio (NLR) sharply increased from preceding major clinical events such the acute stroke noted on day 26 (having most likely occurred on the 21st day) and the patient’s worsening respiratory function during day 32. It is further worth noting the pattern of changes in the rest of his haematology tests showing a rise in NLR and fall in lymphocyte to C-reactive protein (LCRPR) and renal impairment, along with the changes in liver function test results indicating a severe systemic inflammatory response over the first three weeks prior to the onset of acute stroke-like symptoms, peaking during day 22 and day 34. Rather, despite the predictive ability of blood tests and specifically NLR in the context of infection, inadequate specialists were available to consider the overall pattern of changes in his haematology or biochemistry. The significant delay in the patient’s referral to the neurology unit/team and a similar delay in the neuroimaging of the brain both indicate the possibility for underdiagnosis of stroke and other neurological disorders during this pandemic.

## Discussion

We report the first case of COVID-19 presenting with acute onset of severe neurological symptoms (acute ischemic stroke-like symptoms) and radiological changes of intracranial hypertension in Australia with no apparent clinical impact on his visual functions but some on his ability to complete a visuomotor task six weeks post initial hospitalization. The laboratory profile of this patient suggests that a generalized severe immune response to SARS-CoV-2 was apparent early, based on the neutrophil-lymphocyte ratio and CRP, followed by persistent anaemia, leukocytosis and hypoalbuminemia and neurological manifestations during his hospital stay.

COVID-19 has been associated with excessive inflammatory activity, hypercoagulability and hypoxia, leading to both venous and arterial thrombosis. Our case illustrates the importance of critical and in-depth analysis of simple blood tests such as total white cell counts, electrolytes, CRP and coagulation parameters with special attention to changes and patterns of changes such as NLR, CRP and lymphocyte-to-platelet ratio (LPR). A recent meta-analysis noted that NLR values were found to increase significantly in severe cases of COVID-19 (SMD: 2.404,95%; CI: 1.275 to -0.5.50), while LPR values were decreased significantly (SMD: 0.912; 95% CI: -1.275 to -0.550). [11-13]

Our results suggest that a COVID-19-induced hyperimmune response led to significant inflammatory injury to the brain and suspected intracranial hypertension secondary to severe inflammation. It is very likely the onset of acute stroke-like symptoms related to COVID-19 occurred with the first peak of inflammatory markers and NLR during day 21. However, the treating team was unable to pick up the clinical features of cerebral involvement until day 26 due to the tracheostomy and sedated state of the patient. This case illustrates the critical clinical value of regular assessment of NLR, LCRPR (Lymphocyte to CRP Ratio) and LPR (lymphocyte to platelet ratio) which are low-cost prognostic tools available in almost all health services across the world in comparison to sophisticated, expensive investigations including brain MRI.

The peripheral immune response and the behaviour of this patient’s blood biochemistry, particularly their white cell count (neutrophil, lymphocytes, and total white cell count), showed a significant correlation with his recovery process. It is also notable that at several time points, temperature spikes were co-incident with neutrophil spikes, highlighting the need for concurrent analysis and understanding of all types of simultaneously incoming patient data, whether biochemical, immunological, physiological or cognitive parameters (see the attached online supplement spreadsheet for other blood measures including electrolytes).

To date, several international clinical researchers have successfully applied a more integrative artificial intelligence (AI) based approach to data management to assist in clinical decision making in COVID-19 cases. Indeed, Jiang et al. used AI research to determine, based on 53 hospitalized COVID-19 patients, that a mildly elevated liver enzyme alanine aminotransferase (ALT), a high red cell count and muscle pain all serve as good predictive factors of risk of developing acute respiratory distress syndrome (ARDS), common sequelae of COVID-19 [14].

## Conclusions

In conclusion, we argue that this COVID-19 case study further highlights the usefulness of incorporating a more comprehensive framework based around continuous and objective patterns and ratios of blood hematology/biochemistry. AI-based decision making could potentially enable rapid online cloud-secured predictive analytics that can concurrently analyze all types of simultaneously incoming patient data, whether biochemical, immunological, physiological or cognitive parameters. Such an approach will provide the time-stressed clinician with an up-to-date, systematically summarized set of data points for use to assist in choosing treatment and management options. This case also highlights the clinical value of simple, easy-to-use technology such as the Welch RV100-B RetinaVue™ 100 Imager Non-Mydriatic Retinal Camera, visual field perimetry (MRF-n) app in this context.
